# COVID-19 Vaccine Coverage in India: A District-Level Analysis

**DOI:** 10.3390/vaccines11050948

**Published:** 2023-05-05

**Authors:** Sandip K. Agarwal, Maharnab Naha

**Affiliations:** Indian Institute of Science Education and Research (IISER), Bhopal 462066, India

**Keywords:** COVID-19, vaccination, India, vaccine hesitancy, vaccine demand, vaccine supply

## Abstract

India implemented the largest COVID-19 vaccination drive in the world, through which it vaccinated the majority of its population. Lessons from the Indian COVID-19 vaccination experience can be invaluable for other LMICs as well as for preparedness for future outbreaks. Our study is designed to explore the factors associated with COVID-19 vaccination coverage in India at the district level. We used data from COVID-19 vaccination in India combined with several other administrative data to create a unique data set that facilitated a spatio–temporal exploratory analysis by uncovering the factors associated with vaccination rates across different vaccination phases and districts. We found evidence that past reported infection rates were positively correlated with COVID-19 vaccination outcomes. Past cumulative COVID-19 deaths as a proportion of district populations were associated with lower COVID-19 vaccination, but the percentage of past reported infection was positively correlated with first-dose COVID-19 vaccination, which might indicate a positive role of higher awareness created by a higher reported infection rate. Districts that on average had a higher population burden per health centre were likely to have lower COVID-19 vaccination rates. Vaccination rates were lower in rural areas relative to urban areas, whereas the association with literacy rate was positive. Districts with a higher percentage of children with complete immunisation were associated with higher COVID-19 vaccination, whereas low vaccination was observed in districts that had higher percentages of wasted children. COVID-19 vaccination was lower among pregnant and lactating women. Higher vaccination was observed among populations with higher blood pressure and hypertension (which were a few of the co–morbidities associated with COVID-19 infection).

## 1. Introduction

The adverse impacts of the COVID-19 pandemic have been felt in all spheres of human life, with unprecedented challenges to public health and the economy. Herd immunity has been the key to minimise disruptions caused by the pandemic. The sooner the population acquires herd immunity, the sooner human life and activity can be restored to pre-pandemic levels [1,2]. However, relying on the natural process of building up herd immunity would have been slow and would have given room to a prolonged pandemic. Several global leaders and politicians around the world framed their public addresses regarding the pandemic in a war frame with the comfort and confidence that they had a plan in place to emerge victorious in the war against the pandemic [3,4,5]. For governments and policymakers around the world, mass inoculation against COVID-19 has been the biggest weapon to fight the pandemic [6,7].

While scientists have developed vaccines for COVID-19 at unprecedented speed, the process of translating them into manufactured products on a large scale and making them further available to the masses through an uninterrupted and efficient supply chain is an inevitable component of mass inoculation [8,9]. Vaccine shortages due to limited manufacturing capacity or due to inefficiencies in the supply chain would adversely affect the vaccination drive. In addition, a successful vaccination campaign requires much more than the availability of a safe and effective vaccine. Availability of a vaccine does not mean that people will rush to become inoculated. Introduction of a new vaccine demands rigorous research surrounding psychological, social and political aspects to assess public trust in the vaccine as much as it demands scientific rigorous evidence of safety and efficacy of the vaccine [10,11]. As a result, the observed vaccination outcome is the interaction between the supply of and the demand for the vaccine, where inadequate demand, supply or both may lead to low vaccination rates. In the context of vaccine demand, a delay in acceptance or refusal of vaccination despite availability of vaccination services has been defined as vaccine hesitancy [12]. In 2019, the World Health Organization (WHO) declared vaccine hesitancy as one of the top ten threats to public health. Timely addressing vaccine hesitancy can avert an adverse public health outcome [13].

COVID-19 vaccination rates have been the lowest among low- and middle-income countries (LMICs) [14,15,16]. As most LMICs relied on other vaccine-producing nations for their supplies, vaccination against COVID-19 was slow in the LMICs [17,18]. In addition, a host of complicated demand-driven issues surrounding vaccine hesitancy may also have contributed to low vaccination rates, as has been indicated in vaccine hesitancy or uptake surveys [14,19,20,21,22]. India is one of the few LMICs that produced COVID-19 vaccines domestically [23]. In the past decade, India has stood out as one of the largest vaccine producers in the world, with a share of around 60% of vaccine supplies to UNICEF [24,25,26,27,28,29]. The COVID-19 vaccination campaign in India has been one of the largest in the world [30,31]. In comparison to other LMICs, the proportion of the Indian population and the pace at which they were vaccinated against COVID-19 has been phenomenal [32,33,34].

In this article, we analyse COVID-19 vaccination rates in India. Our research is built upon a novel data set that has been created by combining several administrative data sets and COVID-19 data sets for India. While researchers have analysed the district-level COVID-19 infections and fatalities [35,36], as far as we know, our study is the first one to provide a comprehensive analysis of COVID-19 vaccination rates for all of the districts of India across different phases of the COVID-19 vaccination campaign in the country. Most existing research studies that have analysed the regional variation in the observed COVID-19 vaccination rates in India are mostly confined to state-level analyses or analysis of a particular state or district for early phases of the vaccination [37,38,39,40]. In addition, we also draw the association between various demographic, socio–economic and health variables with the observed vaccination rates in the district. Our research makes important contributions to the field of public health research by improving our understanding of factors that are associated with COVID-19 vaccination rates. Our findings from the Indian COVID-19 vaccination drive can significantly contribute to the learning of other LMICs regarding vaccination programs in LMICs [41]. Our article is organised as following: Section 2 lays down the background for vaccination in India in general, followed by specific details related to the COVID-19 vaccination campaign, including the timeline for COVID-19 vaccination in India. Section 3 describes the materials and methods. Section 4 presents the results, and Section 5 discusses the findings. Section 6 concludes the paper.

## 2. Background

India has a history of public immunization programs since its colonial times [42]. India set up an immunisation programme in the 1970s (which was later renamed the Universal Immunization Program (UIP)) with the objective of providing life-saving vaccinations, and it played a critical role in lowering India’s child and neonatal mortality and morbidity rates. While anti-vaccine sentiments in India have never been an organised anti-vaccine movement, unlike in the West, prior to the UIP, during British India and after independence, a few prominent Indian leaders who had mass appeal among the people were apprehensive of the safety and efficacy of vaccines or questioned the compatibility of vaccines with their religious beliefs. The UIP also met with criticism from a section of elitists on account of being a relatively costly program for a poor country such as India [42]. During the first decade of the UIP’s operation, various immunisation coverage levels reached 70–85%; it has declined since then [43]. However, UIP successfully eliminated polio and maternal and neonatal tetanus from India in 2015 [44]. India’s confidence in achieving its ambitious target to vaccinate its population of 1.3 billion people against COVID-19 was driven by its strong domestic sector vaccine production and its past successful experience with large public immunization programmes [30].

**Figure 1:**
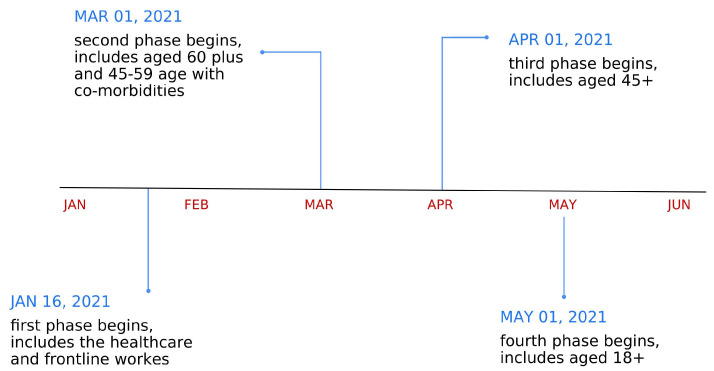
Timeline of COVID-19 vaccination phases in India.

The COVID-19 vaccination drive in India began on 16 January 2021 with two approved vaccines. The COVID-19 vaccination being voluntary in nature left ample scope for vaccine hesitancy. The COVID-19 vaccination drive in India prioritised the section of the population at the highest risk of infection, which was the rationale for the phased rollout of the vaccines [45]. The timeline of this is depicted in Figure 1.

Phase-1 COVID-19 vaccination started on 16 January 2021 and went on until 28 February 2021 and allowed first-dose vaccination of frontline workers who were actively involved in containing the spread of the pandemic. The frontline workers included health workers, security staff and people who provided essential services to the general public. We earmarked the period before Phase 1—from the beginning of COVID-19 in India in March 2020 up to 15 January 2021—as the pre-vaccination phase or Phase 0, though technically there was no Phase 0 in the vaccination drive. During Phase-2, which was implemented between 1 March to 31 March 2021, population aged 60 years and above was eligible for fist vaccine dose. In addition, individuals aged 45 years and above with comorbidities such as hypertension, diabetes, HIV infection, etc., were also eligible. Phase 3, which ran between April 1 and 30, made all individuals aged 45 years and above eligible for first dose of COVID-19 vaccination. All adults aged 18 years and above were eligible for the first dose of vaccine from 1 May 2021 onwards, which was the last and final phase of COVID-19 vaccination in India. Any individual eligible for vaccination during a particular phase remained eligible for vaccination in all subsequent phases.

**Figure 2:**
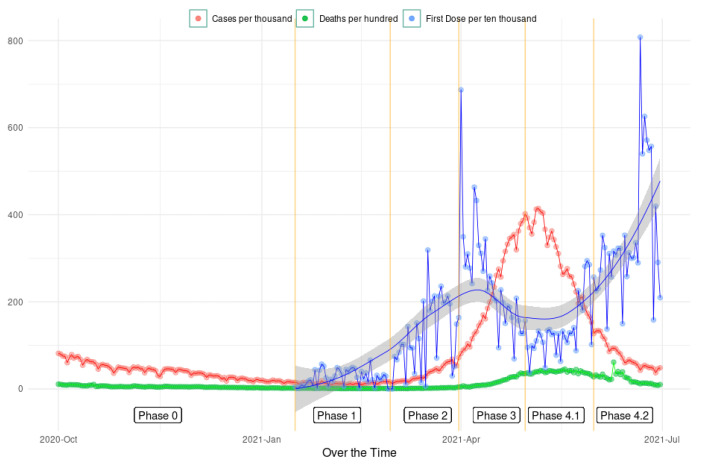
Reported COVID-19 infections, COVID-19 deaths and first doses of COVID-19 vaccination in India across different phases.

Figure 2 plots all of India’s reported COVID-19 cases, reported COVID-19 deaths and first doses of COVID-19 vaccine administered over time during the different vaccination phases. While Phase 0 started in March 2020, which is the beginning of COVID-19, Phase 0 has been truncated in the figure to focus on the vaccination phases. Moreover, we only considered the first two months of Phase 4, which were May and June 2021, primarily because this coincided with the deadly second wave of COVID-19 in India, as can be seen in Figure 2.

## 3. Materials and Methods

We used data from various secondary sources to create a unique data set at the district level that allowed us to investigate the heterogeneity of vaccination rates across different districts and vaccination phases. We obtained district-level data for daily cumulative COVID-19 first-dose vaccination numbers, daily cumulative COVID-19 deaths and daily cumulative reported caseload numbers from the Data Development Lab [46]. From the above cumulative data, the total number of first-dose COVID-19 vaccinations, total reported COVID-19 caseload and total reported COVID-19 deaths were calculated for every district during all phases by taking the difference between the last dates of two consecutive phases. The calculated numbers were divided by district-level population data from the Harvard Population Database (Dataverse) [47] to arrive at district-level first-dose COVID-19 vaccination rates, total reported COVID-19 caseloads or infection rates and total reported COVID-19 death rates as percentages of the district-level populations. The Harvard Population Database (Dataverse) provides district-level population data for India for 2020.

Table 1 summarizes the descriptive statistics of phase-wise COVID-19 variables. IR% is the infection rate, defined as the reported number of infections during a particular phase as a percentage of the district population. PIR% and PDR% are the past cumulative infection rate and past cumulative death rate, respectively, as percentages of the district population aggregated over all the prior vaccination phases. For Phase 1, PIR% and PDR% are the total reported COVID-19 infections and total reported COVID-19 deaths in a district since the first case of COVID-19 was reported in India in March 2020 until 15 January 2021 (the day prior to the beginning of Phase 1 COVID-19 vaccination). We call this, similarly, PIR% and PDR% for Phase 4 Month 2 (i.e., June 2021), which are the the total reported COVID-19 infections and total reported COVID-19 deaths, respectively in a district until 31 May 2021 expressed as a percentage of the district population.

We used the data from the Rural Health Statistics (RHS) for the year 2020–2021 from the Health Information Management System (HMIS) portal of India to construct a measure of district-level health infrastructure. RHS data provided us with the number of sub-health centres, primary health centres and community health centres in each district, which were converted into an equivalent number of sub-health centres by using the equivalence ratio provided in the RHS report (6 and 24 sub-health centres for a primary and a community health centre, respectively) [48]. Two measures of health infrastructure variables were constructed using the total equivalent number of sub-health centres in each district. The first one measured the average population burden on a sub-health centre, which was computed by dividing the population in that district by the number of equivalent sub-health centres in that district. A second variable was constructed to measure the geographical density of health infrastructure by dividing the number of equivalent sub-health centres by the geographical area of the district (i.e., the number of sub-health centres per square km). This can be an indicator of the average distance from the health facility, as the higher the density in a district, the more likely that the average distance travelled to access the health centre is smaller.

We also included district-level data from the fifth round of the National Family and Health Survey (NFHS) conducted between 2019–21 in order to take into account district-specific heterogeneity in the health status and its association with COVID-19 vaccination outcomes. NFHS is an important database that tracks health status over time and has had administrative units in India collecting data every four to five years since 1992–1993. Some of the notable variables from NFHS-5 that we included in our analysis include child and maternal health (including immunization and nutrition), years of schooling, family planning, etc.

District-level demographic and socio–economic variables, including percentage of rural population, literacy, gender, social and religious minorities and population density from the 2011 census of India were also included. In addition, we also included an indicator for aspirational districts. The government of India introduced the aspirational districts programme in early 2018 to reduce inter-district and inter-state disparity by bringing the state and the central governments together on various developmental schemes through healthy competition among the districts to achieve improved targeted health, education and infrastructure outcomes. The programme identified the 117 most-underdeveloped districts as aspirational districts based on a composite index that captured deprivation in health, education and infrastructure domains [49].

Table 2 summarizes the descriptive statistics of district-level variables that are time invariant. We created a district-level data set for our analysis using the variables from various data sources as described above.

We employed ordinary least squares (OLS) regression to find the associations between COVID-19 vaccination and other relevant variables from our data set. Our primary dependent variable was the percentage of the population vaccinated with a first dose of COVID-19 vaccine during every time period (denoted by phases and Months 1 and 2 for Phase 4). The independent variables that we used in our regression included phase-wise vaccinations, cumulative reported infections and cumulative deaths as a percentage of the population. In addition, we also used time-invariant (vaccination phase), district-specific demographic, socio–economic and health variables as independent variables in our regressions. We estimated a separate OLS regression for each time period (or phase). For all regressions, we used state-fixed effects to control for unobserved state effects. We also used clustered robust standard errors, which were clustered at the state level.

## 4. Results

The results of phase-wise OLS regression are shown in Columns 1 to 5 of the regression Table 3 and Table 4 with P1 to P4 as the vaccination phase and subscripts 1 and 2 with Phase 4 as the first and second months, respectively, of Phase 4.

All regression models included four COVID-19-specific explanatory variables that varied across districts and phases—PIR, PDR, (PIR/PDR) and IR, along with demographic and socio–economic variables and measures of health infrastructure. The results have been tabulated in Table 3. Standard errors were clustered at the state level.

In Table 4, we also include other district-level health and education indicators along with all other variables that were used in Table 3.

## 5. Discussion

COVID-19 vaccination in India was provided free of charge to all. Despite this, people might not have perceived it to be costless due to several opportunity costs and unobserved psychological costs in addition to lack of trust in the vaccine that might have led to vaccine hesitancy. Vaccine hesitancy could have been a major driver of low vaccine demand and subsequently low vaccination rates despite the availability of the vaccine. Availability of a vaccine does not mean that people will rush to become inoculated. The observed vaccination rate is an outcome of the complex interaction between the supply as well as the demand for the vaccine rather than one single component alone.

The reported infection rate in a district, while likely to be indicative of the actual infection rate, was also confounded with COVID-19 testing efforts. Districts that implemented more rigorous COVID-19 testing were also likely to report a higher number of COVID-19 cases compared to districts that had lower COVID-19 testing rates. On the contrary, unlike reported infection rates, COVID-19 fatalities were more closely associated with the actual infection rate and were unlikely to be influenced by COVID-19 testing efforts. Both COVID-19 reported infection rates and death rates were likely to be correlated with vaccination rates, and hence we included these variables in all regression models in Table 3 and Table 4.

For all phases, we observed vaccination rates to be positively correlated with past cumulative reported infection rates, PIR, as a percentage of the total district population. The above association might have been driven by higher COVID-19 awareness in districts with higher reported infection rates that could be either due to higher testing efforts or the prevalence of an actual higher infection rate. We also included reported infection rates for the current phase IR%, which stood out to be positive and significant only during the first month of Phase 4. The initial first month of Phase 4 coincided with the deadly second wave of COVID-19 infections in India, which might have pushed up the demand for vaccination. There was a negative association between past COVID-19 deaths (PDR) and vaccination in any district, though this was significant for Phases 1 and 4 only. PDR% was defined as the past cumulative death rate out of the total district population. On the contrary, the association between vaccination rate and past COVID-19 deaths as the proportion of the past infection rate (PDR/PIR) was positive and statistically significant for the first and fourth phases. The opposite signs of COVID-19 death variables when measured as a proportion of the population and when measured as a proportion of the infection rate might be capturing two different phenomena. At unchanged reported infection rates, while a higher PDR in a district indicated higher fatalities among the population, a higher (PDR/PIR) in a district indicated higher COVID-19 deaths for unit infection in that district. A higher number of COVID-19-linked deaths might have pushed up the demand for COVID-19 vaccination.

In the above discussion, we attempted to think of possible reasons for the observed relationship between past and current reported infections and deaths, which tell a demand-side story. However, it cannot be undermined that COVID-19 infections and deaths were also used as critical inputs in vaccine allocation to different districts. Vaccination rates were likely to be lower in districts that were constrained due to a limited supply of vaccines. However, vaccination rates during an entire phase are likely to be less volatile than during a particular week or day due to volatility in the vaccine supply. Therefore, while not impossible, it is less likely that a district would have faced severe supply constraints due to a short supply of vaccine during the entire vaccination phase. Nonetheless, we would not attribute lower vaccination rates to low demand only, but rather a combination of both demand- and supply-side factors [45,50].

The health infrastructure burden was captured by the average population served by each sub-health centre in a district. A lower health burden is indicative of better health infrastructure and is negatively associated with vaccination rates across all vaccination phases, and the coefficient was significant except during Phase 1. Since Phase 1 targeted vaccination of health workers and frontline workers, it is not surprising to not find a significant correlation between health infrastructure and vaccination rates across districts. Health infrastructure density measured the average number of sub-health centres per square kilometre in a district. During the vaccination phases used in our analysis, COVID-19 vaccines were available on demand at vaccination sites only, which implied that individuals had to travel to the vaccination centres to become vaccinated. While a lower density is likely to be an indicator of a greater distance from the health centre for an average person in a district, we found an inverse and significant correlation between health infrastructure density and COVID-19 vaccination rates in early Phase 4. The beginning of Phase 4 COVID-19 vaccination in India coincided with the deadly second wave (as can be seen in Figure 1), with high COVID-19 infections and deaths along with multiple localised lockdowns, which affected densely populated areas more severely—also adversely affecting the vaccination campaign. Given that the population density and health infrastructure density are positively correlated, the above finding might not be surprising.

With respect to different demographic and socio–economic variables, we did not find them to explain heterogeneity in the vaccination rates across districts during different vaccination phases except for Phase 4. Vaccine attitudes are dynamic and evolve over time and space. Therefore, it was possible that these correlations could have emerged only during the fourth phase of vaccination, when India was hit by the deadly second wave of COVID-19 and the country witnessed the worst situation in the entire pandemic [50,51,52,53,54,55,56].

Vaccination was negatively correlated with a higher proportion of rural population in a district. There is anecdotal evidence that suggested high vaccine hesitancy among the rural population during the beginning of the fourth phase, which aligns with the “co-incidence dragon” (post hoc ergo propter hoc: after this, therefore because of this) in the literature [12,57]. As stated earlier, rollout of COVID-19 vaccination in India prioritised the section of the population most susceptible to COVID-19 infection and fatalities. This section of the population included the elderly and those with comorbidities who had been vaccinated with the first dose of COVID-19 vaccine but were yet to be vaccinated with the second dose of COVID-19 vaccine. It is important to mention that COVID-19 vaccines in India and elsewhere were mostly two-shot vaccines. High efficiency would require that both doses of vaccine be administered in order to provide protection against fatal infection. During the second COVID-19 wave, which coincided with the early fourth phase of vaccination, several deaths occurred among vaccinated people who were mostly vaccinated with the first dose of COVID-19 vaccine. Anecdotal evidence suggests that this led to the belief that vaccines were deadly and caused deaths, giving way to co-incidence dragon bias, particularly in rural areas [58,59,60,61]. Nonetheless, we cannot assert that co-incidence dragon bias led to a reduction in vaccination numbers during the fourth phase. Given the severity of the second wave of COVID-19, it cannot be ruled out that vaccine distribution would have been prioritised to favour urban and more densely populated regions to contain the spread of infection, which also could have adversely affected the vaccination drive in rural areas.

Vaccination was positively correlated with higher literacy rates. COVID-19 vaccination campaigns in India required an online registration to become vaccinated during the period of our analysis. In addition, during the period of our analysis, the registration platform was only accessible in English. Therefore, appointment registration for COVID-19 vaccination required access to a smart phone and internet along with English literacy. These factors could have partially driven the relationship between literacy and vaccination in addition to differences in attitude and perception towards the vaccine shaped by literacy.

Districts with higher concentrations of marginalized communities—Scheduled Caste (SC) and Scheduled Tribe (ST)—also had lower vaccination rates. Lower vaccination rates were observed among concentrations of Muslim populations during Phase 2. However, during the second month of Phase 4, vaccination rates were relatively higher in districts with higher Muslim populations. For Muslims, Ramadan, the holy month of fasting, coincided with the first month of Phase 4. Therefore, it might have been that vaccination uptake was higher among Muslims once Ramadan concluded [62,63,64].

We found vaccination rates to be associated with selected maternal health indicators. Higher maternal protection against neonatal tetanus was positively correlated with COVID-19 vaccination. We found negative significant correlation between vaccination rates and pregnancy rates (between the ages of 15 and 19 years) and percentages of children exclusively breastfed. Women of childbearing age were eligible for vaccination during the fourth phase of vaccination, but a lack of scientific evidence and clear communication surrounding potential side-effects of the vaccine on a pregnant or a breastfeeding woman might have driven this finding [65,66,67].

Similarly, we also found COVID-19 vaccination to be associated with child health indicators. We found positive and significant correlation between complete immunisation of children and COVID-19 vaccination rates during Phases 3 and 4 of the vaccination [68,69]. However, for given complete immunisation levels, the correlation between polio vaccination and COVID-19 vaccination was negative. It is important to note that unlike all other vaccines in the complete immunisation schedule, which are invasive, polio vaccines are oral vaccines. Hence, the negative correlation between COVID-19 vaccine with polio vaccine might be indicative of aversion towards invasive medical procedures, which could have built some hesitancy around the COVID-19 vaccine, which was also invasive. Moreover, adult vaccination is not common in India as in any developing country, unlike child immunisation, which might also have built resistance against COVID-19 vaccines [70]. Lower vaccination was also observed in districts with higher proportions of wasted children.

A positive and significant correlation between COVID-19 vaccination rate and the proportion of the population on medication for blood pressure during Phase 2 and Phase 3 was observed. Since high blood pressure and hypertension were some of the identified comorbidities associated with COVID-19 infection, it may have driven the COVID-19 vaccination rate higher. We also found a positive correlation between health insurance coverage and vaccination rates. Vaccination rates were also higher in aspirational districts during the initial first two phases of vaccination.

We compiled district-level data from various secondary sources on COVID-19 vaccination, COVID-19 infections and COVID-19 deaths, which we combined with demographic and health data in order to facilitate heterogeneity analysis across districts and phases. We would like to state that the observed relationships in our analysis should not be interpreted as causal relationships between vaccination rates and other explanatory variables. There might be some element of causality in our analysis, which would be invaluable if it could be identified; a causal analysis is limited due to the unavailability of requisite data. We also understand that there might have been measurement errors, particularly with respect to COVID-19 variables, due to significant under-reporting; yet our analysis is defensible unless there were systematic errors in measurement of variables. However, given all these limitations, we make our best effort to bring the several pieces of data together to provide an exploratory analysis of COVID-19 vaccination in India.

## 6. Conclusions

Our study attempted to explain the variation in the observed COVID-19 vaccination rates across districts in India during the first few months of the COVID-19 vaccination drive. With vaccination decisions being a complex interplay between the demand and supply of a vaccine, it is difficult to identify the causal factors that might influence observed vaccination rates, as the observed vaccination outcome could indicate lack of supply, demand or both. Therefore, our exploratory analysis provides suggestive evidence surrounding observed vaccination rates, based on which we suggest possible reasons that might have led to the observed empirical relationships.

We used district-level data from the first few months of the COVID-19 vaccination drive in India, which we combined with several other administrative data to create a unique data set that facilitated heterogeneity analysis across different vaccination phases and districts. We found evidence of past reported infection rates positively correlated with first doses COVID-19 vaccination. Past cumulative COVID-19 deaths as a proportion of district population was associated with lower COVID-19 vaccination, but the percentage of past cumulative reported infections was positively correlated with COVID-19 vaccination. A higher population burden per health centre was likely to indicate a lower COVID-19 vaccination rate. Districts with larger rural populations had lower vaccination rates, whereas the association between COVID-19 vaccination and literacy rate was positive. Higher percentages of fully immunized children were positively associated with COVID-19 vaccination, whereas vaccination rates were lower in districts with higher percentages of wasted children. COVID-19 vaccination was lower among pregnant and lactating mothers. Higher COVID-19 vaccination rates among populations with higher blood pressure and hypertension (some of the comorbidities of COVID-19 infection) was observed.

Our research makes an important contribution to the area of vaccine research in the context of LMICs by using data from India, which implemented the largest COVID-19 vaccination drive in the world. We uncovered several associations between vaccination rates and health and demographic variables that provide insights regarding COVID-19 vaccination and vaccination in general that can be used to design future research and investigations surrounding vaccination in LMICs. 

## Figures and Tables

**Table 1:** Phase-wise summary statistics of COVID-19 variables.

Phase 1	Obs	Mean	Median	Max	Min	St. Dev
FDV %	729	0.95	0.77	5.75	0.13	0.65
IR %	629	0.04	0.01	1.48	0.00	0.18
PIR %	629	0.64	0.44	6.05	0.00	0.68
PDR %	629	0.01	0.00	0.11	0.00	0.01
(PDR/PIR) %	625	1.18	1.05	4.99	0.00	0.84
**Phase 2**	**Obs**	**Mean**	**Median**	**Max**	**Min**	**St. Dev**
FDV %	729	2.97	2.48	17.12	0.21	2.05
IR %	629	0.05	0.01	1.48	0.00	0.13
PIR %	629	0.674	0.450	7.007	0.000	0.75
PDR %	629	0.01	0.00	0.11	0.00	0.01
(PDR/PIR) %	626	1.17	1.04	4.99	0.00	0.82
**Phase 3**	**Obs**	**Mean**	**Median**	**Max**	**Min**	**St. Dev**
FDV %	729	6.02	5.04	23.40	0.40	3.88
IR %	629	1.11	0.72	9.66	0.00	1.22
PIR %	629	0.73	0.48	7.36	0.00	0.82
PDR %	629	0.01	0.00	0.12	0.00	0.01
(PDR/PIR) %	626	1.11	1.03	4.27	0.00	0.73
**Phase 4—Month 1**	**Obs**	**Mean**	**Median**	**Max**	**Min**	**St. Dev**
FDV %	729	3.35	2.62	21.01	0.20	2.62
IR %	629	0.64	0.42	8.18	0.00	0.69
PIR %	629	1.84	1.18	17.02	0.00	2.01
PDR %	629	0.01	0.01	0.13	0.00	0.015
(PDR/PIR) %	626	0.59	0.56	2.41	0.00	0.36
**Phase 4—Month 2**	**Obs**	**Mean**	**Median**	**Max**	**Min**	**St. Dev**
FDV %	729	8.42	6.82	41.56	0.94	5.90
IR %	629	2.48	1.70	20.30	0.00	2.56
PIR %	629	0.18	0.06	2.61	0.00	0.29
PDR %	629	0.02	0.01	0.16	0.00	0.02
(PDR/PIR) %	626	0.79	0.72	3.77	0.00	0.49

**Table 2:** Demographic and health data variables.

Var	Des	Obs	Mean	Median	Max	Min	St. Dev
Rural	% Rural households	729	74.1	80.4	100.0	0.0	21.4
Literacy	% Population literate	729	62.1	61.8	88.7	28.8	10.5
Muslim	% Population Muslim	729	12.3	7.2	98.5	0.3	17.0
SC	% Population SC	729	14.6	15.5	50.2	0.0	9.3
ST	% Population ST	729	19.3	4.8	99.4	0.0	28.4
Pop_den	Population density	729	1145	421	82,845	1.5	4780
Sex ratio	Sex ratio of total population	729	1022	1016	1332	755	72.7
	(females per 1000 males)						
Pop_15	% Population below age 15 years	729	26.4	25.3	50.6	16.0	5.3
Insurance	% Households with any usual	729	40.2	35.7	97.8	1.2	23.05
	member covered under a health						
	insurance/financing scheme						
Water_impr	% Population living in	729	93.6	96.9	100.0	41.2	9.1
	households with an improved						
	drinking-water source						
Pre-primary	% Children aged 5 years who	729	12.7	9.8	52.9	0.0	10.3
	attended pre-primary school						
	during the school years 2019–2020						
Tetanus_mother	% Mothers whose last birth	729	91.1	92.6	100.0	55.1	6.2
	was protected against						
	neonatal tetanus						
Vax_full_child	% Children age 12–23 months	729	76.3	77.5	100.0	0.0	15.7
	fully vaccinated based on						
	information from either						
	vaccination card or						
	mother’s recall						
Vax_polio_child	% Children aged 12–23 months	729	80.0	81.9	100.0	0.0	14.8
	vaccinated with 3 polio doses						
Sterilization	% Female sterilization	729	34.9	34.0	76.5	1.1	18.9
School_women	% Women with 10 or more	729	40.4	39.3	88.2	13.6	14.1
	years of schooling						
Early_marriage	% Women age 20–24 years	729	20.6	18.6	80.9	0.0	12.7
	married before 18 yrs						
Early_pregnant	% Women age 15–19 years	729	6.5	4.9	91.9	0.0	6.5
	who were already mothers						
	or pregnant at the time of						
	the survey						
HT_female	% Females with elevated blood	729	21.5	21.1	42.1	8.5	5.2
	pressure (BP) or taking						
	medicine to control BP						
Breastfed_child	% Children under age of 6 months	729	41.3	55.0	94.0	0.0	33.5
	exclusively breastfed (%)						
Wasted_child	Children under 5 years who were	729	18.5	18.0	48.0	4.5	6.5
	wasted (weight for height)						
SHC_burden	Population served by an	727	2768	2557	21045	179	1574
	average sub-heath centre						
SHC_density	Average number of sub-health	727	0.3	0.2	21.8	0.0	1.1
	centres per sq. km						
Adist	Dummy for aspirational district	729	0.16	0.00	1.00	0.00	0.37

**Table 3:** Regression table without the NFHS data variables.

	Dependent Variable: FDV%				
	(P1)	(P2)	(P3)	(P4_1)	(P4_2)
PIR%	0.67 ***	1.16 ***	2.04 ***	0.19 ***	1.54 ***
	(0.05)	(0.10)	(0.15)	(0.06)	(0.15)
PDR%	−12.69 ***				−80.44 ***
	(3.28)				(15.65)
(PDR/PIR)%	0.09 **			−0.87 ***	1.25 ***
	(0.04)			(0.24)	(0.47)
IR				0.81 ***	
				(0.20)	
SHC_burden		−0.0002 ***	−0.0003 ***	−0.0002 ***	−0.001 ***
		(0.0000)	(0.0001)	(0.0001)	(0.0001)
SHC_density					−4.69 ***
					(1.03)
Rural				−0.03 ***	−0.03 **
				(0.01)	(0.01)
Literacy			0.08 ***	0.03 ***	0.09 ***
			(0.01)	(0.01)	(0.02)
Pop_den			0.0002 ***		0.001 ***
			(0.0001)		(0.0002)
Sex ratio		0.002 **	0.003 **		
		(0.001)	(0.001)		
SC					−0.06 **
					(0.02)
ST	0.01 ***				
	(0.001)				
Muslim		−0.01 **			0.04 ***
		(0.005)			(0.01)
Constant	−0.03	−0.32	−5.83 ***	4.36 ***	2.54
	(0.13)	(1.03)	(1.70)	(0.99)	(2.16)
State FE	Yes	Yes	Yes	Yes	Yes
Observations	625	629	629	626	626
AIC	604.79	2030.32	2561.77	2279.99	3195.05
BIC	751.23	2190.3	2726.2	2439.81	3372.62
R${}^{2}$	0.64	0.70	0.78	0.66	0.72
Adjusted R${}^{2}$	0.62	0.68	0.77	0.65	0.71

*** *p*-value < 0.001, ** *p*-value < 0.01.

**Table 4:** Regression table with the NFHS data variables.

	Dependent Variable: FDV				
	(P1)	(P2)	(P3)	(P4_1)	(P4_2)
PIR%	0.61 ***	1.09 ***	2.03 ***	0.34 ***	1.53 ***
	(0.05)	(0.10)	(0.15)	(0.09)	(0.14)
PDR%	−11.16 ***			−26.52 **	−72.47 ***
	(3.20)			(10.58)	(15.42)
(PDR/PIR)%	0.08 **	0.02	0.09	−0.38	1.33 ***
	(0.04)	(0.09)	(0.16)	(0.29)	(0.47)
IR%				0.77 ***	
				(0.19)	
SHC_burden		−0.0002 ***	−0.0003 ***	−0.0002 ***	−0.001 ***
		(0.0000)	(0.0001)	(0.0001)	(0.0001)
SHC_density					−3.81 ***
					(1.02)
Rural				−0.04 ***	
				(0.01)	
Literacy					0.12 ***
					(0.02)
Pop_den					0.001 ***
					(0.0002)
Sex ratio					−0.01 ***
					(0.002)
SC					−0.07 ***
					(0.02)
ST	0.01 ***		−0.01 ***		
	(0.001)		(0.005)		
Muslim		−0.01 ***			0.04 ***
		(0.005)			(0.01)
Pop_15	−0.02 ***				
	(0.01)				
Insurance	0.01 ***			0.02 **	0.05 ***
	(0.002)			(0.01)	(0.02)
HT_female		0.07 ***	0.10 ***		
		(0.01)	(0.02)		
Pre-primary		−0.02 ***			
		(0.01)			
School_women			0.05 ***		
			(0.01)		
Early_marriage					0.09 ***
					(0.02)
Early_pregnant					−0.13 ***
					(0.05)
Breastfed_child				−0.01 ***	
				(0.002)	
Tetanus_mother					0.05 *
					(0.03)
Vax_full_child			0.05 **		0.08 **
			(0.02)		(0.04)
Vax_polio_child			−0.06 **		−0.10 **
			(0.02)		(0.04)
Wasted_child				−0.05 ***	
				(0.01)	
Sterilization	−0.01 ***				
	(0.002)				
Water_impr		0.02 **			
		(0.01)			
Adist	0.15 ***	0.31 **			
	(0.05)	(0.14)			
Constant	0.21	−1.72 *	−1.10	5.40 ***	−2.50
	(0.29)	(0.98)	(0.87)	(0.94)	(3.96)
State FE	Yes	Yes	Yes	Yes	Yes
Observations	625	626	626	626	626
AIC	571.81	1996.14	2527.83	2255.96	3174.98
BIC	736.01	2164.84	2696.52	2429.1	3379.19
R${}^{2}$	0.66	0.71	0.79	0.68	0.74
Adjusted R${}^{2}$	0.64	0.69	0.78	0.66	0.72

*** *p*-value < 0.001, ** *p*-value < 0.01, * *p*-value < 0.05.

## Data Availability

The data sets generated and/or analysed during the current study are available from the corresponding author on reasonable request.
